# Early Diagnosis of Liver Metastases from Colorectal Cancer through CT Radiomics and Formal Methods: A Pilot Study

**DOI:** 10.3390/jcm11010031

**Published:** 2021-12-22

**Authors:** Aldo Rocca, Maria Chiara Brunese, Antonella Santone, Pasquale Avella, Paolo Bianco, Andrea Scacchi, Mariano Scaglione, Fabio Bellifemine, Roberta Danzi, Giulia Varriano, Gianfranco Vallone, Fulvio Calise, Luca Brunese

**Affiliations:** 1 Department of Medicine and Health Sciences “V. Tiberio”, University of Molise, 86100 Campobasso, Italy; mariachiarabrunese@libero.it (M.C.B.); antonella.santone@unimol.it (A.S.); avella.p@libero.it (P.A.); a.scacchi@studenti.unimol.it (A.S.); f.bellifemine@studenti.unimol.it (F.B.); g.varriano@studenti.unimol.it (G.V.); gianfranco.vallone@unimol.it (G.V.); luca.brunese@unimol.it (L.B.); 2 HPB Surgery Unit, Pineta Grande Hospital, 81030 Castel Volturno, Italy; biancopaolo@virgilio.it (P.B.); fulvio.calise@libero.it (F.C.); 3 Department of Radiology, Pineta Grande Hospital, 81030 Castel Volturno, Italy; mariano.scaglione@pinetagrande.it (M.S.); roberta.danzi@pinetagrande.it (R.D.); 4 Department of Medicine, Surgical and Experimental Science, University of Sassari, 07100 Sassari, Italy

**Keywords:** colorectal liver metastases, radiomics, liver metastases prediction, radiology, cancer radiomics

## Abstract

Background: Liver metastases are a leading cause of cancer-associated deaths in patients affected by colorectal cancer (CRC). The multidisciplinary strategy to treat CRC is more effective when the radiological diagnosis is accurate and early. Despite the evolving technologies in radiological accuracy, the radiological diagnosis of Colorectal Cancer Liver Metastases (CRCLM) is still a key point. The aim of our study was to define a new patient representation different by Artificial Intelligence models, using Formal Methods (FMs), to help clinicians to predict the presence of liver metastasis when still undetectable using the standard protocols. Methods: We retrospectively reviewed from 2013 to 2020 the CT scan of nine patients affected by CRC who would develop liver lesions within 4 months and 8 years. Seven patients developed liver metastases after primary staging before any liver surgery, and two patients were enrolled after R0 liver resection. Twenty-one patients were enrolled as the case control group (CCG). Regions of Interest (ROIs) were identified through manual segmentation on the medical images including only liver parenchyma and eventual benign lesions, avoiding major vessels and biliary ducts. Our predictive model was built based on formally verified radiomic features. Results: The precision of our methods is 100%, scheduling patients as positive only if they will be affected by CRCLM, showing a 93.3% overall accuracy. Recall was 77.8%. Conclusion: FMs can provide an effective early detection of CRCLM before clinical diagnosis only through non-invasive radiomic features even in very heterogeneous and small clinical samples.

## 1. Introduction

Liver metastases are a leading cause of cancer-associated deaths in patients affected by colorectal cancer (CRC) [1,2]. Concerning the best patient care, a multidisciplinary strategy has been developed to treat CRC, involving surgeons, oncologists, radiotherapists, and radiologists [3,4,5]. The team is essential to provide data that are required for any practicing surgeon to guide the best patient care [6,7,8,9,10].

To date, the R0 surgical resection of liver metastases is still considered the best treatment for curative purposes [11]. In recent years, the new parenchyma sparing techniques and the accurate ultrasound-guided liver resections allow even more wide surgical indications and the possibility of further surgery also in case of recurrence [12]. The accurate and early diagnosis of all intrahepatic lesions prior to surgical resection is necessary to best plan the whole oncological management [6,13].

Despite the evolving technologies in radiological accuracy, the radiological diagnosis of Colorectal Cancer Liver Metastases (CRCLM) is still a key point [14].

The standard of clinical practice detects liver lesions through tri/quadriphasic CT scan associated with MRI only in selected cases [6,7,13,15,16,17,18].

It should be underlined that the most challenging clinical picture is represented by liver micrometastases, which are not detectable with the standard radiological protocols, becoming evident only in a later time [19,20].

So, an earlier and more accurate diagnosis of liver micrometastases could provide more appropriate treatment and surveillance strategies, which implies a more intensive follow-up [15,16,17,20,21].

Currently trained physicians visually assess medical images for the detection, characterization, and monitoring of diseases. On the other hand, the application of Artificial Intelligence (AI) methods automatically evaluates imaging data and provides quantitative, rather than qualitative, assessments of radiographic characteristics [22].

Some studies had already investigated computed models to achieve an earlier detection of liver metastases, but they were limited to machine learning methods in a short follow-up time frame [15,17,21].

Those studies showed that the most important limit of AI and machine learning is the need of a huge cohort of cases to achieve a satisfying sensitivity and specificity rate [23].

In this paper, considering previous successful experiences achieved in other settings [24,25,26,27,28,29], we propose to use for the first time Formal Methods (FMs) to investigate liver parenchyma. We aim to early detect liver metastases by computing non-invasive shape-based radiomic features from CT images when they are still undetectable using the standard protocols [30,31].

Our secondary end points are:

- To demonstrate the effectiveness and reliability of FMs also in a long follow-up time frame and in a small population cohort.
- To assess the effectiveness and reliability of FMs imaging detection also after hepato-Biliary surgery.

## 2. Materials and Methods

### 2.1. Dataset

We retrospectively reviewed the CT scan data of 30 patients collected between January 2013 and June 2021 at the Pineta Grande Hospital Castel Volturno, Caserta, Italy. All patients included in the study underwent CT scan at our centre using SOMATOM^®^ Definition Flash (dual-source 128-slice CT scanner), Siemens Healthcare, Erlangen, Germany. A total of 21 patients were enrolled as a case control group (CCG). CCG included “healthy” patients who underwent triphasic CT scan for other causes without any radiological or anamnestic evidence of cancer disease or chronic illness. Any evidence of underlying liver disease excluded patients from the CCG. Benign liver lesions, defined as angiomas and hepatic cysts, were not considered an exclusion criterion. The case group (CG) included 9 patients affected by CRC who would develop liver lesions within 4 months and 8 years, while 7 patients developed liver metastases after primary staging before any liver surgery, and 2 patients were enrolled after R0 liver resection. The evidence of any liver lesion at first CT scan was considered as exclusion criteria. The inclusion criteria were:

- Evidence of CRC diagnosed at CT scan confirmed with histopathological exam;
- Patients who performed the first CT scan and follow-up exams at our centre, in order to set the protocol on the same scan;
- No evidence of liver lesion at the moment of primary diagnosis or at follow up after surgery;
- Findings of previous liver surgery in metachronous patients already treated with surgical approach.

The exclusion criteria were:

- Evidence of synchronous liver lesions at first CT scan;
- Underlying liver disease in both groups;
- CT scan performed in other centres or with other type of scan setting.

### 2.2. Image Acquisition and Segmentation

**Figure 1:**
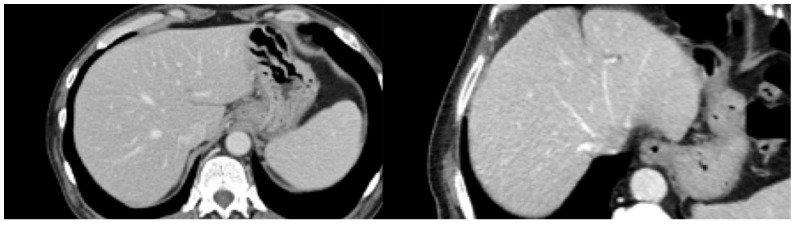
Healthy liver (**left**), metastatic liver (**right**).

The image acquisition phase regarded the CT scan portal phase, while all other phases were excluded. The program analyzed images without symbols or other patterns detectable at the naked-eye view, as shown in Figure 1. Regions Of Interest (ROIs) were manually defined, checked, and reviewed on a 3D-Slicer by trained physicians [32].

**Figure 2:**
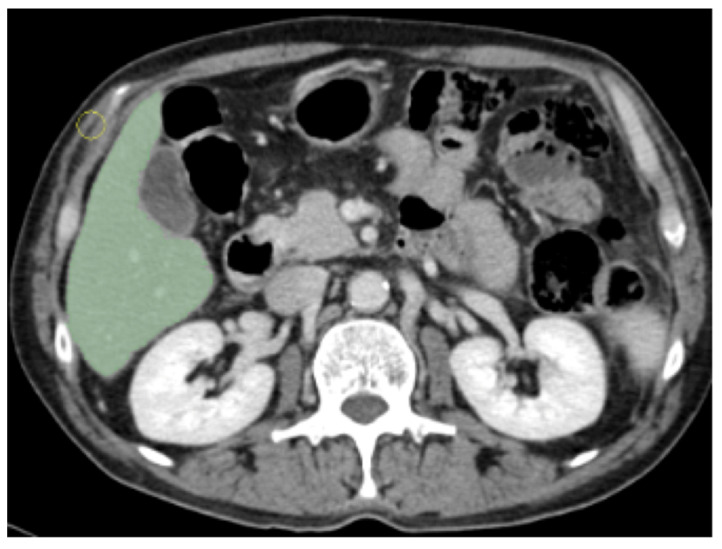
Example of manual segmented Region Of Interest (ROI).

We excluded the portal vein, the inferior vena cava branches, and the gallbladder, as shown in Figure 2. Our approach granted the inclusion of the whole liver parenchyma and biliary structures.

### 2.3. Radiomics Feature Extraction and Reduction

The process of ROI segmentation was performed slice by slice in the whole portal phase series. Successively, radiomic features were extracted through “Pyradiomics” (Boston, MA, USA), an extension of the 3D Slicer software [33]. Pyradiomics is a Python library that allows the file conversion of radiological images into numerical features to extract data not achievable at “naked-eye view”. Feature extraction was conducted on a mean of 70 (56–92 CI) ROI/patient, and successively, a feature selection has been performed. Radiomic features were analyzed by Weka [34]. Weka (General Public License, New Zealand) is an open-source software that uses algorithms of machine learning and data mining to identify the most discriminating features [34]. It is a simple way to apply machine learning methods to a CSV dataset [34], and it allows easily analyzing results. Through these methods, AI can predict new data behaviors. Weka [34] imports patients ID in a dataset, all radiomic features are scheduled in rows, while clinical data are scheduled in columns. Then, it is possible to select the most discriminating features related to “healthy” and “metastatic” livers. The final step is called feature reduction or feature selection. The features reduction outputs resulted in 22 features divided into the 5 classes defined by the radiomic standards (Table 1) [35]. Below, we provide the list of features selected as “significant”, but not “redundant” [35].

### 2.4. Formal Methodology

**Figure 3:**
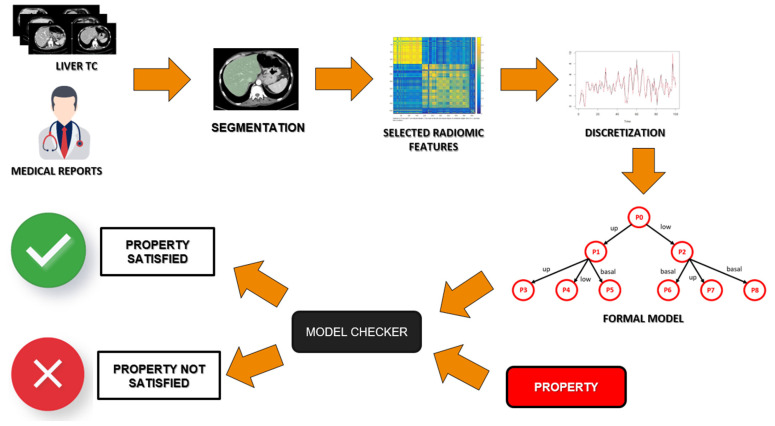
Schema of the formal verification approach.

FMs are based on Milner’s algebra [36], which is a mathematical logic currently used to build a model that gathers all the information of the state of health of a single patient. Then, these models must be verified through a formal verification agent, the Model Checker [37]. In Figure 3, a schema of the used approach is shown. Starting from the radiological images, these are segmented and discretized according to some selected radiomic features. Once these features are translated into a formal model of the health status of a patient, the Model Checker agent will verify, on that model, if the property representing the disease is present.

The formal model contains the health status of a patient that is represented by different radiomic features. As a matter of fact, each single feature value is computed by a single slice of the radiological exam: for this reason, the discrete values of each feature can be different between healthy patients and sick patients. Thanks to Formal Methods, researchers can highlight these differences and use them for a second virtual opinion.

Once the model was built, it is necessary to define, also with the help of the radiologist, a certain state of health: in FMs, the disease is described by some “properties” or “formulas”, needing to be used for the classification task. The properties logically combine similarities between patients with the same state of health to better emphasize the state of health and find some patterns. In this study, these properties will set the rules to recognize “healthy” or “metastatic” livers.

The Model Checker checks if a property is verified on each patient model [38,39]. This logic rule includes a logical-temporal reasoning [37] that is needed to link different CT slices, so it guarantees a multi-slice approach. The model checker returns “True” or “False” depending on whether the property is satisfied on the patient model. All the above steps of the formal verification are summarized in Figure 3.

### 2.5. Outcome Extraction

The outcomes used to determine the reliability of the classifier were:

- True Positive Rate (TP): Number of metastatic patients correctly classified as “metastatic”;
- False Positive Rate (FP): Number of healthy patients wrongly classified as “metastatic”;
- False Negative Rate (FN): Number of metastatic patients wrongly classified as “healthy”;
- True Negative Rate (TN): Number of healthy patients correctly classified as “healthy”.

Considering the above definition, different performance metrics were calculated, as shown in the following subsection:

- Precision: correct assignment to the class of positives;
- Recall: the completeness of the assignment to the class of the positives;
- Accuracy: the fraction of correctly classified cases.

## 3. Results 

**Figure 4:**
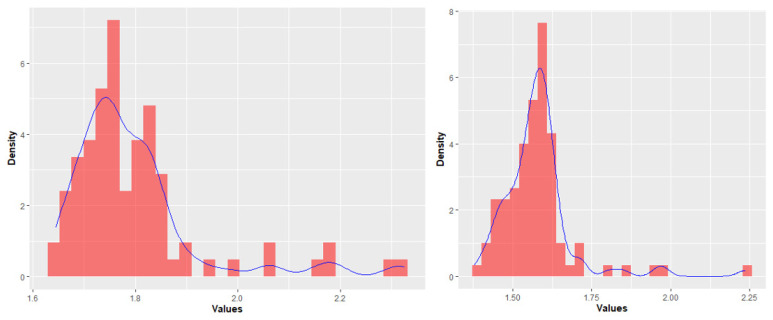
Statistical distributions of “metastatic patients” (**left**) and “healthy patients” (**right**). The blue line shows the trend of distribution of feature values.

The most discriminating features’ classes were: First-Order features (FIRST), Grey-Level Dependence Matrix features (GLDM), Grey-Level Size Zone Matrix features (GLSZM), which were demonstrated to be the most significant considering their distribution between “healthy” and “metastatic” livers. So, we excluded all the remaining features (Figure 4). Uniformity and Entropy were the only features analyzed belonging to first class. Figure 4 shows entropy distributions in different categories of patients.

The precision of our methods is 100%, scheduling patients as positive only if they will be affected by CRCLM, showing a 93.3% overall accuracy. Recall was 77.8%. Table 2 synthetases the clinical reliability of the method. It shows the results in the clinical context rather than in feature classes through the confusion matrix.

The authors are very satisfied with the results obtained: this can be a further step toward the use of radiomics as a tool for the prediction of chronic diseases. The results of this virtual diagnosis for medical doctors are very promising: below, we provide additional accuracy and utility statistics that can help in understanding how powerful the methodology is and what the advantages are over artificial intelligence techniques.

These statistics were derived from the study of Mitchell (Table 3) [40].

## 4. Discussion

Our study showed promising outcomes about the chance to detect liver metastasis from CRC early through FMs analysis of CT radiomic features.

We would underline how FMs achieved a precision rate of 100% allowing us to discern patients who will develop CRCLM with high reliability.

We selected the most discriminating features and properties, so we achieved a global accuracy of 93.3% and a recall rate of 77.8%.

This approach might be advantageous and preferable because when the tumor is not yet detectable through standard protocols, FMs grant the chance to apply a stricter and more effective follow-up. Furthermore, it allows the best management of the health system sources. Comparing our results to other experiences reported in the literature, we found that the FMs reliability is superimposable to the outcomes reported by other authors who investigated the AI application to CRCLM diagnosis [17,19,21]. We started the prediction of CRCLM on the idea of Taghavi et al., which demonstrated the efficacy of machine learning-based analysis of CT radiomics model for the prediction of CRCLM [17].

In conclusion, we can state that both FMs and the other artificial intelligence methods are able to predict the development of liver metastasis [17,19,21].

The limitations of their studies were linked to the large sample size needed to set the machine learning method associated with the need to create a homogeneous sample of cases for the training set and, consequently, the exclusion of patients who had already undergone liver surgery [17]. In order to overcome the need of large samples, FMs were chosen to achieve a high reliability also through a small sample improving precision and sensitivity. This aim can be achieved because FMs are built on properties rationally determined in advance as mathematical formulas by the domain experts (physicians and radiologists). They represent the computer science translation of radiological knowledge; they are applied when liver metastases are not detectable to the human eye. The program applies the correct rule because it learns from physicians and technicians rather than images, as provided by machine learning methods.

Sensitivity and global accuracy may be also improved in FMs, redefining the properties in order to teach the model more information. The outcomes cannot be improved by increasing the number of patients, because the model does not need a training set to work. For this reason, the software is very reliable even with a small sample of cases.

The present study reports for the first time an effective early detection of liver metastases also in a wide time range. Our model predicted the metastases development in a time range of 3–48 months from primary tumor diagnosis. Only one diagnosis was missed in a patient who would develop CRCLM 8 years after CRC diagnosis.

Taking into account that the recurrence rate after CRCLM resections occurs in up to 75% of cases, some authors have already investigated the possibility to early detect the recurrence risk after liver resection [41,42]. Simpson et al. built a reliable pre-operative recurrence/prognosis predictive model that needed the association of CT scan images to clinicopathological variables [42]. We can affirm that we obtained similar results only considering CT images without any clinical data. In our experience, we have correctly predicted recurrence also after surgical liver resection. Radiomic-formal methods-based approaches appear to be promising non-invasive approaches to predict clinical outcome and improve personalized decision making in patients with CRLM. So, an earlier and more accurate diagnosis of liver micrometastases could provide a more appropriate surveillance and treatment strategy, which implies a more intensive follow-up associating CT and MRI.

It allows to best manage the chemotherapy regimens and perform less invasive surgical resections.

## 5. Limitations

ROI were manually defined to ensure the analysis of the entire liver parenchyma. If this process increases sensitivity and accuracy, on the other hand, it requires longer analysis times and the need for a trained team composed of radiologists and computer scientists. It can also be speculated that the sensitivity of our model was hindered by a small sample of cases for different categories. We understand that, but FMs need a small sample of cases to set the model and make it generalizable. Therefore, in this first step, we had the goal to build the model to achieve a reliable early diagnosis and recurrence prediction of CRCLM. Furthermore, our protocol was validated in a single centre cohort, and it was set and reliable only for a single type of CT scan.

## 6. Conclusions

FMs analysis of radiomics features seemed to be reliable and effective to early detect liver metastasis from CRC even in very heterogeneous and small clinical samples. Our study may open the road to more intensive follow-up protocols in patients defined at higher risk by the AI analysis. Further studies should be focused on re-testing our model in a prospective cohort defining ROI in an automatic and more efficient way.

## Figures and Tables

**Table 1:** FIRST, First-Order features; GLDM, Grey-Level Dependence Matrix features; GLCM, Grey-Level Co-occurrence Matrix features; GLRLM, Grey-Level Run Length Matrix features; GLSZM, Grey-Level Size Zone Matrix features.

FIRST	GLDM	GLCM	GLRLM	GLSZM
Entropy	Dependence Entropy	Autocorrelation	High Grey-Level Run Emphasis	High Gray-Level Zone Emphasis
Interquartile Range Mean Absolute Deviation	High Grey-Level Emphasis	Joint Average	Long Run Low Grey-Level Emphasis	Low Grey-Level Zone Emphasis
Mean Absolute Deviation	Large Dependence Low Grey-Level Emphasis	Joint Entropy	Low Gray-Level Run Emphasis	Small Area Low Gray-Level Emphasis
Robust Mean Absolute Deviation	Low Grey-Level Emphasis	Sum Average	Short Run Low Grey-Level Emphasis	________
Uniformity	Small Dependance Low Grey-Level Emphasis	Sum Entropy	________	________

**Table 2:** Clinical output.

**Confusion Matrix**		**Actual Values**	
		Metastatic	Healthy
**Predicted Values**	Metastatic	TP = 7	FP = 0
	Healthy	FN = 2	TN = 21

**Table 3:** Accuracy and utility statistics according to Mitchell [40] to gain more information about the clinical usefulness of the methodology.

Accuracy Statistics	Value	95% Confidence Interval
Sensitivity	77.8%	
Specificity	100.0%	
Positive Predictive Value	100.0%	
Negative Predictive Value	91.3%	
Positive Likelihood Ratio (+Ve)	Inf	
Negative Likelihood Ratio (−Ve)	0.222	
Test Score (or fraction correct) %	93.3%	
Prevalence	30.0%	
**Utility Statistics**	**Rating**	**95% confidence interval**
Clinical Utility (+Ve)	Good	0.778
Clinical Utility (−Ve)	Excellent	0.913

## Data Availability

Data available on request due to restrictions, e.g., privacy or ethical. The data presented in this study are available on request from the corresponding author. The data are not publicly available due to the presence of other sensitive information not included in the study.
